# Ecological context of infant mortality in high-focus states of India

**DOI:** 10.4178/epih.e2016006

**Published:** 2016-03-05

**Authors:** Laishram Ladusingh, Ashish Kumar Gupta, Awdhesh Yadav

**Affiliations:** 1 Department of Mathematical Demography and Statistics, International Institute for Population Sciences, Mumbai, India; 2 Population Council, New Delhi, India; 3 Public Health Foundation of India, Gurgaon, Haryana, India

**Keywords:** Infant mortality, Spatial analysis, Ecological, India

## Abstract

**OBJECTIVES:**

This goal of this study was to shed light on the ecological context as a potential determinant of the infant mortality rate in nine high-focus states in India.

**METHODS:**

Data from the Annual Health Survey (2010-2011), the Census of India (2011), and the District Level Household and Facility Survey 3 (2007-08) were used in this study. In multiple regression analysis explanatory variable such as underdevelopment is measured by the non-working population, and income inequality, quantified as the proportion of households in the bottom wealth quintile. While, the trickle-down effect of education is measured by female literacy, and investment in health, as reflected by neonatal care facilities in primary health centres.

**RESULTS:**

A high spatial autocorrelation of district infant mortality rates was observed, and ecological factors were found to have a significant impact on district infant mortality rates. The result also revealed that non-working population and income inequality were found to have a negative effect on the district infant mortality rate. Additionally, female literacy and new-born care facilities were found to have an inverse association with the infant mortality rate.

**CONCLUSIONS:**

Interventions at the community level can reduce district infant mortality rates.

## NTRODUCTION

India has missed the Millennium Development Goal-IV (MDG-4) target of reducing the infant mortality rate (IMR) to 28 per 1,000 live births by the end of 2015 [1] due to uneven progress among the states of India in reducing infant mortality. The IMR is still alarmingly above the national average in the high-focus states of Uttar Pradesh, Uttarakhand, Bihar, Jharkhand, Chhattisgarh, Odisha, Madhya Pradesh, Rajasthan, and Assam. The 284 districts in these states account for 48.5 percent of India’s population [2]. According to the 2013 Sample Registration System bulletin [1], Madhya Pradesh, Assam, Uttar Pradesh, and Odisha have IMRs above 50 per 1,000 live births, which are the highest rates in the country. The number of districts in these states that have an IMR above the respective state averages was found to be 28 out of 45 in Madhya Pradesh, nine out of 23 in Assam, 34 out of 70 in Uttar Pradesh, and 12 out of 30 in Odisha; moreover, in a few districts, the IMR was above 100 per 1,000 live births. This has drawn the attention of policy-makers and researchers, and led to the launch of the ambitious National Rural Health Mission in 2005. However, even after ten years of the National Rural Health Mission, the pace of the decline in IMR in these high-focus states is unacceptable. The persistently high IMRs in these areas lead to several research questions. Are proximate determinants enough to explain regional disparities in the IMR? Is the regional ecological context a more appropriate target for programmatic interventions? Can increased accessibility of neonatal care further pay off? The objective of this paper was to address some of these questions by re-investigating the determinants of the IMR in the high-focus states of India.

The literature has indicated the importance of socioeconomic status-related factors such as poor living conditions [3]; maternal factors, including age, birth interval, and the experience of child death; birth order; and nutrition [4-6]. Epidemiologists have acknowledged place to be an important determinant of health outcome disparities [7-10], while some have highlighted the importance of geographic factors and underdevelopment in explaining the IMR in the Indian context [11]. Kumar et al. [12] have shown the importance of health programme initiatives in curtailing under-five mortality in the aforementioned states. However, no previous studies have considered the ecological context in determining IMR, which may be the an underappreciated factor in public health.

The present paper seeks to fill this gap in the research by considering the ecological context as a potential determinant of IMR, which makes sense because public health interventions target communities, not individuals. This approach is inconsistent with recent trends in public health that are concerned with improving health conditions and life expectancy in regions with excessively high rates of mortality [13,14]. Keeping in mind that public health interventions must target a few main direct and indirect ecological factors determining infant mortality on the aggregate level, the accessibility of neonatal care facilities was considered in our analysis. Kumar et al. [12] likewise emphasised the close association between accessibility of the health system and infant survival, as has been argued elsewhere by Farmer et al. [15] and Politzer et al. [16]. Keeping this in mind, an attempt was made to quantify the accessibility of neonatal care facilities in the community by evaluating the proportion of public health centers (PHCs) in the district with neonatal care facilities. In the assessment of ecological or macro-level determinants of infant mortality on the aggregate level, many scholars have proposed that underdevelopment has a negative effect on health and other social outcomes [17,18]. Testing Wilkinson’s relative income hypothesis [19], Kawachi & Kennedy [20] have argued that the greater the income gap between the rich and poor, the poorer are the health outcomes. Following their findings, the proportion of households in the bottom wealth quintile at the district level was considered to reflect material inequality instead of district-level household income. The female literacy rate has a trickle-down effect on infant mortality because it has a catalytic influence on healthcare utilization, and the district-level female literacy rate was therefore included in our analysis. Moreover, the proportion of the non-working population at the district level was included as a measure of underdevelopment. The objective of this study was to alert public health and policy planners to the importance of ecological determinants of IMR at the district level. Such an objective cannot be attained by analysing maternal factors alone.

## MATERIALS AND METHODS

### Data

This study drew upon data from three main sources of demographic and health indicators at the district level: District Level Household and Facility Surveys (DLHS; 2007-08) [21], the Annual Health Survey (AHS; 2010-2011) [22], and the Census of India (2011) [2]. The AHS is the only survey specifically designed to provide mortality and fertility indicators at the district level for the nine high-focus states. The AHS was conducted during 2010-2011 and the details of the survey design and instruments used in AHS are available [22]. The DLHS-3 (International Institute for Population Sciences 2012) covered all districts in India as of 2007 and collected data on maternal and child health and the services provided by public health facilities on the district level. The district level health facility information used in the study was obtained from the DLHS-3. The AHS employed similar methods and instruments of data collection. The Census of India (2011) provided data on the population, including the working and non-working population, by age, sex, and place of residence at the district level [2].

The study was based on aggregate data available in the public domain; therefore, no ethical issues were involved.

### Outcome measures

The outcome variable analysed in this study was the district-level IMR, which is the number of infant deaths in a year per 1,000 live births in the same year. The source of the IMR for the 284 districts of the nine high-focus states was the AHS conducted during 2010-2011 [22].

### Explanatory variables

The importance of income and wealth in explaining health outcomes, and particularly infant mortality, was acknowledged in the seminal work of Preston [23] and Filmer & Pritchett [24]. In order to confirm this proposal, the proportion of households in the districts in the lowest wealth quintile was considered as a potential explanatory variable, and this information was available in the report of the AHS [22]. The wealth quintile was determined based on the possession of assets and durable goods in households. Identifying districts with a large concentration of households in the lowest wealth quintile may facilitate intensive mass media and awareness campaigns to promote best practices under the prevailing conditions. The proportion of the unemployed among the population 15 to 59 years of age in these districts was considered in order to assess the effect of interventions such as the Mahatma Gandhi National Rural Employment Guarantee Act (MGNAREGA) [25]. This value was estimated using data from the Census of India [2]. Poverty no doubt has a detrimental effect on child survival, particularly in the first year of life. Lower poverty rates indicate an improved ability to support the younger generation. Thus, the proportion of the unemployed among the population aged 15 to 59 provided empirical evidence regarding the benefit of employment-generation policies in reducing the IMR. The benefit of community educational attainment, particularly among females, is reflected in changes in household behaviour and practices in maternal and child health care [10]. Ladusingh & Singh [10] have highlighted the effectiveness of the educational attainment of household heads in averting infant mortality in northeast India. Following this line of reasoning, the female literacy rate at the district level was one of the explanatory variables included in this study. The demand for paediatric healthcare is as important as the supply, and the level of community education plays an important role in enhancing the uptake of child immunization and the adoption of best healthcare practices. Strengthening adult literacy through the centrally sponsored Saakshar Bharat Programme may be a cost-effective intervention for preventing infant deaths in the under-developed high-focus states. Few studies have considered health facility adequacy as a potential determinant of infant mortality [12]. In our analysis, the accessibility of community health facilities was treated as an important mediating factor for maternal and child healthcare utilization. Considering the importance of accessibility to neonatal care in saving infants from premature death, the proportion of PHCs in the districts providing neonatal care was considered as an explanatory factor. The PHCs are currently being upgraded according to the Indian Public Health Standard in order to meet the healthcare needs of the people.

The explanatory variables discussed above are also to a great extent the factors responsible for the spatial autocorrelation of the incidence of infant mortality in the nine high-focus states in India, as these states share common features regarding healthcare beliefs and practices, as well as accessibility of health facilities, unemployment, and literacy levels.

### Statistical analysis

Descriptive statistics and diagrammatic representations were used to describe outcomes and assess the explanatory variables. Multiple linear regression analysis was used to assess the significance of relationships between the district-level IMR and ecological covariates.

The nine high-focus states considered in this paper have similar sociodemographic and healthcare utilization patterns and are contiguous. Moran’s spatial autocorrelation, denoted by I, was used to assess the similarity of the incidence of infant mortality in neighbouring states. The elements *w_ij_* of W, the weight matrix, denote binary connectivity, with a value of 1 if a district *j* is adjacent to district *i* and is 0 otherwise. Moran’s I statistic is computed as

$$I=\frac{n}{S_{0}}\frac{n\sum_{i}\sum_{j}w_{ij\left(x_{i}-\bar{x}\right)\left(x_{j}-\bar{x}\right)}}{\sum_{j}{\left(x_{i}-\bar{x}\right)}^{2}}$$

where $\bar{x}$ is the mean of the *x* variable, *w_ij_* are the elements of *S_0_* the weight matrix, and is the sum of the elements of the wei­ght matrix:

$$S_{0}=\sum_{i}\sum_{j}w_{ij}$$

The value of I varies from -1 to +1. Positive values of Moran’s I suggest a spatial clustering of similar values. Negative values suggest that high values are found in the vicinity of low values. This metric is very helpful in identifying areas where values of the variable are both extreme and geographically homogeneous. Values near 0 indicate a random spatial pattern [26].

The Moran scatter plot provides a tool for measuring the extent of the autocorrelation among the neighbourhood sections of a society. Anselin et al. [27] described this as spatial lag of the variable on the vertical axis with the original variable on the horizontal axis. Spatial lag refers to the values of neighbouring locations.

## RESULTS

Descriptive statistics regarding the IMR, the percentage of non-workers among population members 15 to 59 years of age, the female literacy rate, the percentage of households in the bottom wealth quintile, and the percentage of PHCs with neonatal care facilities for all nine of the high-focus states are shown in Table 1. The high coefficient of variation (23.1%) reflects high inter- and intra-state variation in the IMR. The high-focus states were at a disadvantage in terms of access to livelihood, with 60.5% of the population in the prime age group of 15 to 59 years not working, a female literacy rate of only 61.9%, widespread poverty with 21.3% of households in the bottom wealth quintile, and 77.6% of the PHCs with neonatal care facilities. It is evident that to reduce IMR, the nine high-focus states should aim to increase employment, improve female literacy, and increase the accessibility of neonatal care.

**Figure 1.**
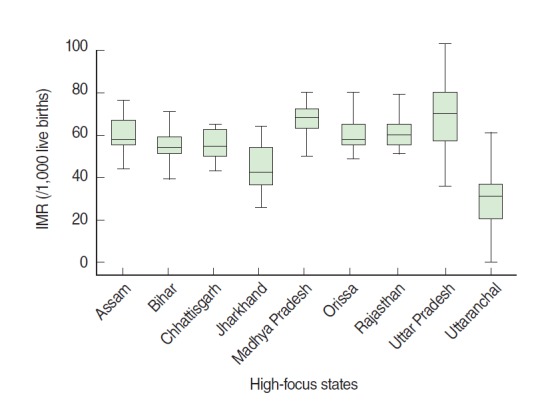
Range and quartile for infant mortality rate (IMR) for high- focus status of India (2010-2011).

Figure 1 comprises the box plots of IMR of the nine states. The median IMR for Uttar Pradesh was 70, meaning that the IMR of 35 districts was above 70; moreover, wide intra-state variability was found, with the IMR ranging from 36 to 103 per 1,000 live births. The median IMR of Madhya Pradesh, with 45 districts, was 68 per 1,000 live births, but the intra-state variation was much larger than that of Uttar Pradesh, ranging from 45 to 80 per 1,000 live births. The IMRs in Assam, Odisha, and Rajasthan, with 23, 30, and 32 districts, respectively, were very close, with median values of 58, 58, and 60 per 1,000 live births, respectively. The median IMR for Bihar and Chhattisgarh was similar at 55, although the intra-state variation was higher in Bihar than in Chhattisgarh. The median IMR for Jharkhand was 42, the second lowest among the nine states after the median IMR of 37 for Uttarakhand. The intra-state variation of the IMR was higher in Jharkhand than in Uttarakhand. The wide inter- and intra-state variation of the IMR in the nine high-focus states in India further underscores the urgency of interventions targeting ecological factors bearing on aggregate district-level infant mortality.

### Moran’s I plot

**Figure 2.**
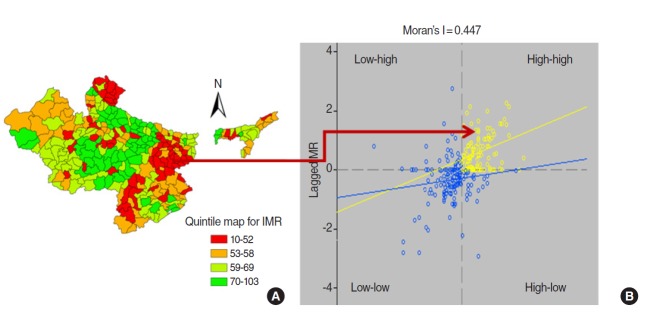
(A) Quintile map for infant mortality rate (IMR) for 2010-2011. (B) Moran’s I scatter plot for IMR for 2010-2011.

A quintile plot (Figure 2A) depicts the location of high-high and low-low patterns of infant mortality. The high-high patterns of the IMR, indicating hot spots, are in districts of the central region (Madhya Pradesh, Uttar Pradesh, and Rajasthan).A scatter plot (Figure 2B) shows the value of the original variable (IMR) on the horizontal axis and the spatial lag-square of the variable on the vertical axis. Both variables are standardized, and the graph is divided into four quadrants; high-high (upper right) and low-low (lower left) indicating positive spatial autocorrelation (being surrounded by locations with similar values). The upper right quadrant shows associations among high values while the lower left quadrant shows associations among low values. Contrastingly, high-low (lower right) and low-high (upper left) correspond to negative spatial autocorrelations.

It was observed that the global Moran’s I statistic of spatial autocorrelation in the IMR between neighbouring areas was 0.4447 (999 permutations; p<0.001), clearly suggesting the presence of a significant spatial correlation among the 284 districts of the high focus states.

The unadjusted and adjusted coefficients and standard errors associated with the ecological factors affecting IMR are shown in Table 2. The percentage of non-workers among persons 15 to 59 years of age had a positive significant effect in explaining variation in IMR (p<0.05).

Both the unadjusted and adjusted effect of female literacy rates at the district level on IMR were significant at p<0.01, with higher female literacy corresponding to lower IMRs. Poverty and income inequality across districts were also considered key factors contributing not only to high IMRs, but also to wide inter and intra-state variation in the IMR. This was confirmed by the positive effect of the percentage of households in the bottom wealth quintile at the district level when other ecological factors are adjusted for. The adjusted effect of the aggregate bottom wealth quintile on IMR was significant (p<0.01). It was found that, after adjusting for other ecological factors, the presence of more neonatal care facilities in PHCs was associated with lower district IMRs (p<0.01). This confirms the need for strengthening PHCs, which are the maternal and paediatric healthcare providers within the community. The significance of the intercept term at p<0.01 indicates that in the nine high-focus states, considerable variation in infant mortality was present even after controlling for these ecological factors, as these factors accounted for 25.8% of the variation in the IMR. Interventions at the individual and community levels are important for the expeditious reduction of the IMR.

**Figure 3.**
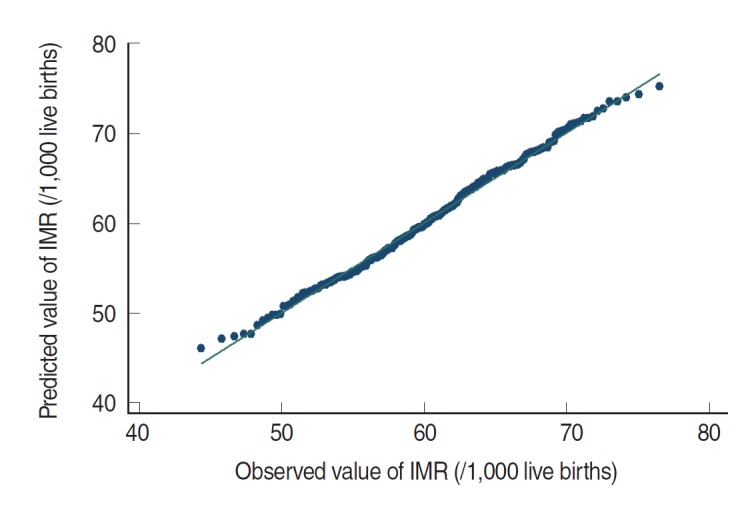
Plot of observed against the fitted infant mortality rate (IMR) (2010-2011).

The plot of the observed IMR against the fitted IMR, shown in Figure 3, confirms the assumption of conformity with normality.

## DISCUSSION

Ecological analyses are important for policy interventions at the regional level, as such analyses explain regional variation more insightfully than explanations focused on individual, maternal, or household-level factors. This paper assessed the significance of ecological factors in explaining regional variation in the IMR. These factors are amendable to favourable changes through public health and community interventions. We suggest that our analysis provides a convincing explanation of some of the reasons behind high IMRs and the presence of wide inter- and intra-state variation in the nine high-focus states in India.

Underdevelopment is a factor responsible for high IMRs in the high-focus states in India. This was reflected in the significant positive effect of the proportion of the non-working population 15 to 59 years of age, which is considered as a proxy measure of underdevelopment. This finding agrees with that of Preston [23], who postulated a strong positive correlation between life expectancy and per capita national income. The positive significant effect of the non-working population at the aggregate level indicates that the high-profile MGNAREGA initiative has yet to employ a sizeable proportion of the working-age population. One of the reasons that female literacy is of prime importance is that it leads to healthier children due to its association with empowerment and emancipation [28]. Female literacy is crucial as the catalyst for the dissemination of good practices in child care, including healthcare utilization. The significant inverse association found between the aggregate female literacy rate and district IMR underscores the importance of improving female literacy through adult literacy programmes. The significance of district-level female literacy in reducing aggregate infant mortality is also a reflection of the sociocultural practices of females in Indian society, and this fact has been ignored by demographers, social statisticians, and population geographers [10]. Income inequality has a negative effect on health outcomes, and it has been overlooked in analysing regional variation in the IMR in India [20]. Wealth quintiles, constructed by assets owned by households, is a close approximation to income [24].The use of the percentage of households in the bottom wealth quintile on the district IMR allowed Wilkinson’s hypothesis to be tested. The finding that higher proportions of households in the bottom wealth quintile were associated with higher IMRs confirmed this hypothesis, in agreement with the similar results of Wilkinson [29,30] and Filmer & Pritchett [24]. Interventions to increase employment can pave the way for reducing income inequality and thereby reducing the IMR. Additionally, for reducing the IMR and meeting the goals of MDG-4, the availability of neonatal care facilities at PHCs is crucial. The inverse association between the proportion of PHCs in districts with neonatal care facilities and district IMRs underscores the necessity of strengthening and increasing accessibility to PHC facilities with neonatal care.

The major limitation of the study is that factors pertaining to sociocultural barriers associated with husbands and mothers-in-law could not be integrated. This limitation could be addressed by carrying out a complementary qualitative study. The main strength of this study is its implications for assessing the feasibility of cost-effective community level interventions, including adult literacy campaigns and upgrading health facilities.

In conclusion, achieving the MDG-4 goals in the nine high-focus states in India may be a realistic target if concerted interventions are made to minimize barriers at the district level. The findings of this study help in theorizing the link between district IMR and its ecological determinants.

## Figures and Tables

**Table 1.** Descriptive statistics of the IMR and its explanatory variables

Variables	No.of districts	Mean	6in	Max	CV
IMR	284	60.6	19.0	103.0	23.1
Percent of non-working population	284	60. a	42.8	73.7	11.3
Female literacy rate	284	61.9	36.9	84.9	15.6
Percent of households in the bottom wealth quintile	284	21.3	1.0	63.7	52.1
Percent of PHCs with neonatal care facilities	284	77.6	53.7	88.9	13.9

IMR, infant mortality rate; Min, minimum; Max, maximum; CV, coefficient of variation; PHC, public health center.

**Table 2.** Unadjusted and adjusted coefficients and SE for the ordinary least squares regression analysis of the effect of ecological factors on the IMR

Variable	Unadjusted		Adjusted	
	Coefficient	SE	Coefficient	SE
Intercept			72.725^**^	12.204
Percent of non-working population	0.190	0.1214	0.287^*^	0.118
Female literacy rate	-0.495^**^	0.0810	-0.365^**^	0.089
Percent of households in the bottom wealth quintile	0.365^**^	0.0716	0.259^**^	0.079
Percent of PHCs with neonatal care facilities	-0.222^**^	0.0757	-0.161^*^	0.072
No. of observations	284			

SE, standard error; IMR, infant mortality rate; PHC, public health center.

* p<0.05,

** p<0.01.

## References

[1] Office of the Registrar General, India SRS bulletin sample registration system [cited 2016 Mar 21]. http://censusindia.gov.in/vital_statistics/SRS_Bulletins/SRS%20Bulletin%20-Sepetem­ber%202014.pdf.

[2] Office of the Registrar General and Census Commissioner, India (2011). Census of India 2011: provisional population totals. Series 1. http://pib.nic.in/prs/2011/latest31mar.pdf.

[3] Manda SO (1999). Birth intervals, breastfeeding and determinants of childhood mortality in Malawi. Soc Sci Med.

[4] Mosley WH, Chen LC (1984). An analytical framework for the study of child survival in developing countries. Popul Dev Rev.

[5] Cleland JG, Van Ginneken JK (1988). Maternal education and child survival in developing countries: the search for pathways of influence. Soc Sci Med.

[6] Das Gupta M (1990). Death clustering, mothers’ education and the determinants of child mortality in rural Punjab, India. Popul Stud.

[7] Kishor S (1993). “May god give sons to all”: gender and child mortality in India. Am Sociol Rev.

[8] Kravdal Ø (2004). Child mortality in India: the community-level effect of education. Popul Stud (Camb).

[9] Deborah Balk D, Pullum T, Storeygard A, Greenwell F, Neuman M (2004). A spatial analysis of childhood mortality in West Africa. Popul Space Place.

[10] Ladusingh L, Singh CH (2006). Place, community education, gender and child mortality in North-east India. Popul Space Place.

[11] Singh A, Pathak PK, Chauhan RK, Pan W (2011). Infant and child mortality in India in the last two decades: a geospatial analysis. PLoS One.

[12] Kumar C, Singh PK, Rai RK (2012). Under-five mortality in high focus states in India: a district level geospatial analysis. PLoS One.

[13] Feinstein JS (1993). The relationship between socioeconomic status and health: a review of the literature. Milbank Q.

[14] James WL, Cossman JS (2006). Does regional variation affect ecological mortality research? An examination of mortality, income inequality and health infrastructure in the Mississippi Delta. Popul Res Policy Rev.

[15] Farmer FL, Stokes CS, Fiser RH, Papini DP (1991). Poverty, primary care and age-specific mortality. J Rural Health.

[16] Politzer RM, Harris DL, Gaston MH, Mullan F (1991). Primary care physician supply and the medically underserved. A status report and recommendations. JAMA.

[17] Deaton A (2003). Health, inequality, and economic development. J Econ Lit.

[18] Drèze J, Sen A (1995). India, economic development and social opportunity.

[19] Wilkinson RG (1997). Socioeconomic determinants of health. Health inequalities: relative or absolute material standards?. BMJ.

[20] Kawachi I, Kennedy BP (1999). Income inequality and health: pathways and mechanisms. Health Serv Res.

[21] International Institute for Population Sciences (2010). District level household and facility survey 2007­08. http://rchiips.org/pdf/india_report_dlhs-3.pdf.

[22] Ministry of Home Affairs, Government of India (2011). Annual health survey. http://censusindia.gov.in/2011-common/AHSurvey.html.

[23] Preston SH (1975). The changing relation between mortality and level of economic development. Popul Stud (Camb).

[24] Filmer D, Pritchett L (1999). The impact of public spending on health: does money matter?. Soc Sci Med.

[25] Department of Rural Development, India Mahatma Gandhi National Rural Employment Guarantee Act-2005 [cited 2016 Mar 22]. http://www.nrega.ap.gov.in/Nregs/.

[26] Moran PA (1950). Notes on continuous stochastic phenomena. Biometrika.

[27] Anselin L, Syabri I, Kho Y (2006). GeoDa: an introduction to spatial data analysis. Geogr Anal.

[28] Shetty A, Shetty S (2014). The impact of female literacy on infant mortality rate in Indian states. Curr Pediatr Res.

[29] Wilkinson RG (1992). Income distribution and life expectancy. BMJ.

[30] Wilkinson RG (1999). Two pathways, but how much do they diverge?. BMJ.

